# Shell shape does not accurately predict self-righting ability in hatchling freshwater turtles

**DOI:** 10.1038/s41598-024-54191-w

**Published:** 2024-02-28

**Authors:** Adam van Casteren, William I. Sellers, Dane A. Crossley, Leah M. Costello, Jonathan R. Codd

**Affiliations:** 1 School of Biological Sciences, University of Manchester, Manchester, M13 9PL UK; 2 School of Natural Sciences, University of Manchester, Manchester, M13 9PL UK; 3 Department of Biological Sciences, University of North Texas, Denton, TX USA

**Keywords:** Biomechanics, Herpetology

## Abstract

Flat hydrodynamic shells likely represent an evolutionary trade-off between adaptation to an aquatic lifestyle and the instability of more rounded shells, thought beneficial for self-righting. Trade-offs often result in compromises, this is particularly true when freshwater turtles, with flatter shells, must self-right to avoid the negative effects of inverting. These turtles, theoretically, invest more biomechanical effort to achieve successful and timely self-righting when compared to turtles with rounded carapaces. This increase in effort places these hatchlings in a precarious position; prone to inversion and predation and with shells seemingly maladapted to the act of self-righting. Here, we examine hatchling self-righting performance in three morphologically distinct freshwater turtle species (*Apalone spinifera*, *Chelydra serpentina* and *Trachemys scripta scripta*) that inhabit similar environmental niches. We demonstrate that these hatchlings were capable of rapid self-righting and used considerably less biomechanical effort relative to adult turtles. Despite differences in shell morphology the energetic efficiency of self-righting remained remarkably low and uniform between the three species. Our results confound theoretical predictions of self-righting ability based on shell shape metrics and indicate that other morphological characteristics like neck or tail morphology and shell material properties must be considered to better understand the biomechanical nuances of Testudine self-righting.

## Introduction

Should a terrestrial animal become inverted they are at an increased risk of predation, exposure, and starvation^1,2^. It is therefore generally assumed that being able to self-right, in a timely and energetically efficient manner, is a fitness related ability^3,4^. Self-righting strategies, interestingly, vary considerably across terrestrial taxa and are heavily influenced by body shape and flexibility^5^.

Testudines are unique among vertebrates in that, with the exception of the neck and tail, the dorsal vertebrae are fused to form the carapace of a bony shell that encloses all the major organs and the shoulder and pelvic girdles; limiting the movements of the limbs^6,7^. An inflexible body and the associated limited limb motion means that Testudine species are prone to inverting, when terrestrially locomoting, and are presented with unique challenges to produce the necessary momentum to self-right^7^.

Once inverted Testudines are generally stable; meaning they are at a low gravitational potential energy. An individual must then add mechanical energy to rotate the shell along their longitudinal axis, until an unstable tipping point is reached and the shell falls back to a stable state, in a prone position with the plastron in contact with the substrate^7,8^. Theoretically, it is easier for turtles with highly domed and rounded carapaces to self-right as they have to raise their centre of mass a smaller amount to reach this unstable tipping point when compared to turtles with a relatively flatter carapace^8^. This means, as is common throughout zoology, turtle shell shape will likely be a trade-off between adaptation for different selection pressures^9^**.** Flatter shells offer superior hydrodynamics, but are thought to confer reduced mechanical protection from predators^9^ and are less protective against the negative effects of inversion such as exposure and dehydration^10^.

There are two distinct kinematic strategies for self-righting in Testudines that are related to overall carapace morphology^2,8^. Testudines with highly domed carapaces, generally self-right by rotating the limbs to generate momentum, shifting the centre of mass, and initiating a shell roll. A strategy seen in terrestrial species like the star (*Geochelone*)*,* leopard (*Stigmochelys*) and Madagascan (*Astrochelys*) tortoises and in some semi-aquatic species like the North American box turtles (*Terrapene*)^1,2,7,8,11^. Alternatively, species with flatter and often more streamlined carapaces employ a different tactic. The neck is extended and then used to provide a vertical force which flips the shell over^8^. This neck-based strategy is characteristic of aquatic and semi-aquatic turtles like the mud (kinosternids), pond (emydids), snapping (chelydrids) and soft-shelled (trionychids) varieties^7,8^.

Given the importance of self-righting in Testudines to survival^4^, previous work has focused on assessing the efficiency of this behaviour in terms of the time to complete the action and/or predicted energetic input. Time is often used as an easily measurable metric to characterise the fitness advantage of self-righting^4,10,12–14^. The assumption is that a faster righting time conveys a greater fitness as the individual more rapidly escapes the supposed negative effects of inversion. Such approaches have demonstrated correlations between self-righting time and shell shape. In a range of terrestrial *Testudo,* an increase in the domed shape of the carapace was correlated with a reduction in the time to self-right^13,15^. Similarly, in a comparison of freshwater turtle species those with an increased shell sphericity were capable of quicker righting times. Although, interestingly, intraspecies variation in shell shape did not account for any significant variation in self-righting abilities^10^. Theoretical calculations have also linked shell shape to the effort required to self-right in two species of giant Galápagos tortoises. Chiari et al.^1^ showed that more convex carapaces reduce the amount of input energy needed to self-right^1^, a result in line with predictions^8^. However, it must be noted that such findings are estimates and purely theoretical, the authors did not analyse the kinematics or forces produced by Galápagos tortoises during self-righting. Based on correlations, like those highlighted above, some have even suggested using indices of shell shape as a proxy for self-righting ability and assigning performance optima across the Testudine phylogeny^16^.

While time to self-right and carapace curvature may act as reasonable proxies for self-righting ability, there are currently only a handful of studies aimed at understanding how the biomechanical or energetic costs of self-righting may be reduced by morphological adaptations of the carapace or body. Without such research, it is extremely hard to test if the correlation between shell shape and self-righting performance is causal or not. One recent study by Rubin et al.^2^ found that successful self-righting events in the pink-bellied side-necked turtle (*Emydura subglobosa*) were accompanied by greater moments, that produced faster rolls when compared to unsuccessfully attempts. Head positioning and the yaw of the body had little influence on whether the self-righting action was successful or not. In this way, it would appear that successful self-righting had little to do with the shell and was facilitated by increases in neck length. Ruhr et al.^7^ also found that proportionally, the time to self-right and the biomechanical effort of this action increased with age in snapping turtles (*Chelydra serpentina*). This is despite remarkably similar shell shape metrics across all ages. The optimized self-righting of younger turtles was not a consequence of shell-shape but attributed to the proportionally longer necks of younger individuals. Both these studies seem to indicate that shell shape confers less of a biomechanical advantage than theoretical or ecological studies would indicate. However, current biomechanical studies of self-righting in turtles have remained very intraspecific, where shell shape remains somewhat uniform. This limits our ability to test the influence of shell shape on self-righting biomechanics. Added to this, these studies have also focused exclusively on mature turtles and do not include any hatchlings in the analysis.

Hatchlings are in a crucial stage of their life history where they face proportionally higher mortality compared to adult turtles that can expect a high rate of adult survival and long-life^17,18^. Due to this high mortality, any adaptive traits that can help improve the survival of juveniles will likely be subjected to intense selection^4,18^. The situation becomes even more pertinent for juvenile aquatic turtles. Hatchlings of many species are regularly destabilized and inverted during the transitions between terrestrial and aquatic mediums^3,4^. Given these conditions, many authors have considered self-righting ability in aquatic juvenile turtles as a trait that is directly relevant to survival^3,4,19,20^, although explicit evidence supporting a link between self-righting time and juvenile survival has, as of yet, not been found^21^. Understanding the biomechanical costs of self-righting and how these may be mitigated by morphological traits may help understand the importance of self-righting as a survival strategy for hatchling turtles.

**Figure 1:**
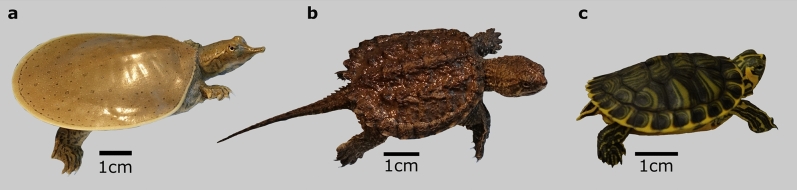
Representative hatchlings of each freshwater species. (**a**) *Apalone spinifera* (spiny softshell turtle), (**b**) *Chelydra serpentina* (common snapping turtle) and (**c**) *Trachemys scripta scripta* (yellow-bellied slider).

Here we investigate the biomechanics of self-righting in hatchlings of three freshwater turtle species (Fig. 1) endemic to the continental United States of America: *Apalone spinifera* (spiny softshell turtle)*, Chelydra serpentina* (common snapping turtle) and *Trachemys scripta scripta* (yellow-bellied slider)*.* These three species offer somewhat of a natural experiment to explore the influence of various morphological features on the biomechanics of self-righting.

*A. spinifera* is a softshell turtle, its shell morphology reflects a specialised adaptation to aquatic environments and burrowing in soft sand. It possesses a shell that is low and circular, lacking any external plates, being covered instead in a smooth undivided leathery skin^22–24^ (Fig. 1a). They also exhibit reduced plastron bones meaning that their underside is less protected from potential attacks^23^. This overall shell morphology should, theoretically, place hatchlings of *A. spinifera* at an interesting juxtaposition. Low mechanical protection from a reduced shell places them at greater risk of predation^10^ and would indicate that they must self-right quickly to escape. Yet their flatter shells should necessitate the need for a larger perturbation of the centre of mass to achieve successful self-righting^8^. We hypothesise that the flatter more hydrodynamic shell shape of *A. spinifera* should increase the biomechanical effort needed to self-right when compared to the other two species. Like *A. spinifera*, the plastron is much reduced in *C. serpentina*, offering similarly lowered level of mechanical protection^14,19^. However, hatchlings of *C. serpentina* possess an armoured and more rounded carapace and a long tail that has been shown to be used during self-righting events^19^ (Fig. 1b). With such morphological traits *C. serpentina* would seem better placed to self-right quickly and with minimal effort. As such we hypothesis that this species would demonstrate the lowest biomechanical input to self-right. *T. s. scripta* has an armoured and rounded shell comparable to that of *C. serpentina* ^22^. Notably though, hatchlings of *Trachemys* possess a full plastron^25^, ossification begins before hatching and is complete quickly within 107 days post hatching ^26^, meaning members of *Trachemys* can rely on their underside being afforded substantial bony protection. *T. s. scripta* also has a much smaller tail, that is not thought to be used in self-righting (Fig. 1c). This would imply that *T. s. scripta* may employ a more passive response to predators^14^ and rely less on quick and efficient self-righting to escape predation, although individuals would still face the non-predatory negative effects of inversion. With a rounded shell but seemingly a reduced reliance on self-righting, we hypothesis that hatchlings of *T. s. scripta* will lie between *A. spinifera* and *C. serpentina* requiring a middling amount of effort to self-right.

## Methods

### Animals

Hatchlings were purchased from a commercial reptile dealer. A total of 17 turtles were used in this study, (*A. spinifera* N = 6, 85 trials*, C. serpentina* N = 6, 63 trials and *T. s. scripta* N = 5, 22 trials)*.* Turtles were individually housed in small 1 L plastic containers filled with water and were allowed to feed on a diet of Mazuri dry Turtle food (Mazuri, PMI Nutrition International, Brentwood, MO) ad libitum*.* Tanks were kept in a climate-controlled room kept at a constant 30 °C and all experiments were also conducted within this room. Turtle husbandry and experimental procedures were carried out in accordance with an animal-care protocol (no. 11-007), approved by the University of North Texas Institutional Animal Care and Use Committee.

### Morphometrics

Before experiments began hatchlings had linear measurements of their shells taken using digital callipers (Duratool, model D02264, Premier Farnell, Leeds, UK), the mass of each turtle was obtained during the experiments from the force plate (details below). Neck lengths were only made on *A. spinifera* and *C. serpentina*. They were measured opportunistically, using a ruler when the neck was fully extended. The timid nature of *T. s. scripta* meant the turtle was quicker to retract the head and keep the neck retracted when in the presence of human experimenters. This meant we were unable to take reliable neck measurements from this species.

The linear measurements were then used to calculate two indices of shell shape: sphericity index (SI) and flatness index (FI). These normalized indices can be used to investigate if shell shape is influencing self-righting performance. SI and FI are defined in Eqs. (1)^27^ and (2)^28^.

1 $${\text{SI}} ={\left(\frac{W \times H}{{L}^{2}}\right)}^\frac{1}{3}$$

2 $${\text{FI}}= \frac{L+ W}{2H}$$

*L* and *W* are maximum carapace length and width respectively and *H* is the shell height.

### Self-righting experimental setup

The experimental set up consisted of a force plate (Advanced Mechanical Technology, Inc. (AMTI), model HE6X6 A1854, Watertown, MA, USA) which was used to record the vertical reaction force exerted by a hatchling during self-righting. The force was measured at 1200 Hz using the AMTI NetForce program (version 3.6.0). To record the self-righting movements a camera (Sony Cyber-shot RX10 III, Sony Corporation, Japan) mounted on a tripod was used, recording at 100 fps for the duration of the trials.

Each trial followed a set procedure. First, to sync the video data with the force plate data a golf ball was placed on the force plate for *circa* 5 s and then removed, providing a known reference point in each medium. To obtain hatchling mass, a hatchling was then placed onto the force plate for a period of *circa* 5 s or for as long as possible without any movements occurring. This gave a period of stable force plate readout which can then be averaged to obtain the hatchlings mass. Following these initial steps, the hatchlings were then inverted onto their backs and the vertical forces were recorded during self-righting. The hatchlings were continually inverted until a maximum of ten viable self-righting events (see below) were recorded or the turtle showed any overt signs of fatigue. In some cases, the turtle did not actively self-right and in these cases a maximum period of ten minutes to collect self-righting events was allowed. Experimenters were present in the experimental room for trials involving *A. spinifera and C. serpentina*. This is primarily because self-righting was prolific and the turtles needed constant inverting. However, *T. s. scripta* would only self-right without the experimenters present in the room. In these trials’ experimenters would exit the room and re-enter after a self-righting event had taken place to invert the turtle again.

### Data processing

Data from the force plate was filtered with a 10 Hz, 2nd order double pass Butterworth low-pass filter, using the SciPy Python package^29^. The video data was then used to locate the timings of viable self-righting events. An event was deemed viable if the turtle started from a stable position on its back and contact between the experimenter’s hand the turtle had ended before the self-righting manoeuvre began.

**Figure 2:**
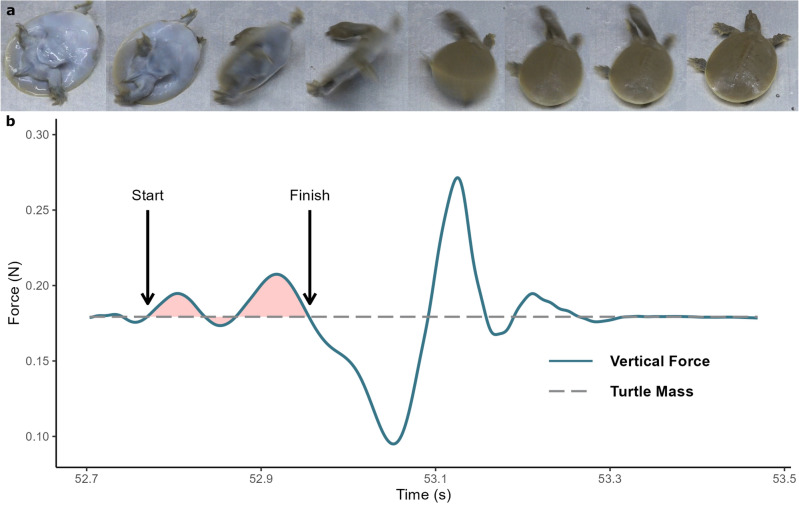
(**a**) Still pictures depicting the self-righting action by *A. spinifera.* (**b**) A representative trace from a single trial of a turtle demonstrating the vertical ground reaction forces measured during a self-righting event. Arrows indicate the start and finishing timing of the self-righting event and shaded area represents impulse to self-right.

Once a self-righting event was identified in the video the timing was used to locate the start point in the filtered force plate data, where the ground reaction force increased above the turtle’s weight (Fig. 2). The end point of a self-righting event was defined as the point when the animal no longer has to actively flip (Fig. 2). The vertical force provided by the turtle’s body weight was then subtracted from the total force to ensure that only the impulse required to perform the self-righting action is analysed (shaded area Fig. 2).

Once an appropriate self-righting event had been identified, we used the equations presented in Ruhr et al.^7^ to calculate the biomechanical costs of self-righting. The impulse (*J*) was calculated as the area under the force–time curve using Eq. (3).

3 $$J= \sum {F}_{t}{\Delta }_{t}$$

*F*_*t*_ represents the instantaneous vertical force and *∆*_*t*_ is the time increment, which is this study was 1/1200 s. The kinetic energy equivalent (KEE) can then be calculated from this impulse using Eq. 4, assuming a start from rest, so that the initial momentum is zero. Where *M*_*b*_ represents turtle body weight. It must be remembered that KEE is not an actual measurement of energetic expenditure but rather a model, based on the gain in kinetic energy that a particle at rest would achieve if subjected to this impulse, that allows an estimation of the energetic cost of an action. KEE has been used in this study as directly measuring the energetic costs of swift movements like self-righting is problematic. Options might include measurements of lactic acid build-up following anaerobic activity, but these are often complex and invasive procedures^30^. It was therefore decided to use force and time measurements to estimate the mechanical energy needed to self-right as has been done in previous studies^7^.

4 $${\text{KEE}}= \frac{{J}^{2}}{2{M}_{b}}$$

Using KEE and the time to complete a self-righting event (*t*_*flip*_) it is possible to calculate the mean power-output equivalent (PE) for that specific action, using Eq. (5).

5 $${\text{PE}}= \frac{\Delta {\text{KEE}}}{{t}_{flip}}$$

To assess the self-righting energetic efficiency, the height-change equivalent (HE) is calculated. This is essentially KEE normalized to turtle mass and carapace width as defined by Eq. (6), where *g* is the gravity acceleration (9.81 m s^−2^). Lower values of HE represent higher self-righting energetic efficiency^7^.

6 $$\ {\text{HE}}= \frac{\Delta {\text{KEE}}}{{M}_{b}\cdot g\cdot W}$$

### Data analysis

The experimental set up composed of 17 turtles. A total number of 170 observations were conducted. The hypothesis for testing is whether the biomechanical outputs of self-righting: KEE, PE and HE and self-righting time are different between the three species of turtle.

All statistical tests were run in R (version 4.1.3; R Core Team, 2022). Linear Mixed Effects Model (LMM) were fitted to our data. In all cases the dependant variable was the biomechanical output, the fixed independent variable was the species of turtle, and the individual was set as the random effect. An ANOVA (type II) followed by post hoc analyses comparing estimated marginal means (EMMs) were performed to conduct pairwise comparisons between the three different species^31^. To ensure the residuals of the model were normally distributed all the continuous dependant variables, except time, were log-transformed. Full models and outputs can be found in supplementary material S1.

### Ethics

Turtle husbandry and experimental procedures were carried out in accordance with an animal-care protocol (no. 11–007), approved by the University of North Texas Institutional Animal Care and Use Committee.

## Results

### Morphology

**Figure 3:**
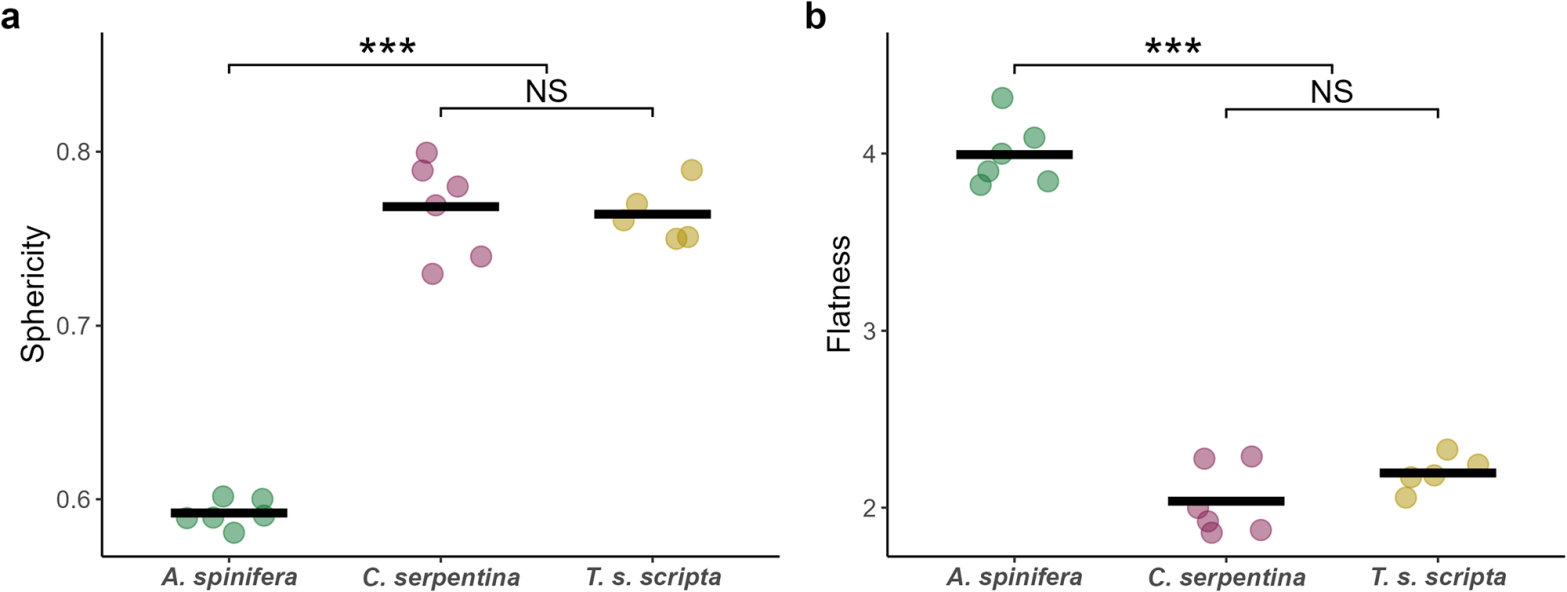
Shell shape indices (**a**) Sphericity, where higher values indicate a more spherical shell and (**b**) Flatness, where higher values indicate a flatter shell. *A. spinifera* demonstrates markedly different shell morphology compared to the *C. serpentina* and *T. s. scripta*. Each point represents an individual, solid bars represent means, *** = p < 0.001 and NS indicates no significant difference.

Linear measurements (Table 1) were used to calculate normalized shape indices of sphericity and flatness. These indices highlight significant shape difference in the hatchling species of this study. *A. spinifera* shells demonstrated significantly lower measurements of sphericity (One-way ANOVA F_2,14_ = 237.1, p < 0.001) and increased flatness (One-way ANOVA F_2,14_ = 155, p < 0.001) while there were no significant differences in shell shape between *C. serpentina* and *T. s. scripta* (Fig. 3).

**Table 1:** The average mass and linear shell dimensions of the hatchlings used in self-righting experiments.

Species	Mass (g)	Shell length (mm)	Shell width (mm)	Shell height (mm)	Neck length (mm)
*A. spinifera*	16.35 (1.28)	55.00 (1.85)	48.18 (0.62)	12.97 (0.77)	4.05 (0.16)
*C. serpentina*	12.04 (0.83)	35.39 (1.63)	33.35 (0.84)	16.35 (1.22)	2.90 (0.20)
*T. s. scripta*	11.04 (0.95)	37.77 (1.02)	37.39 (0.62)	17.34 (0.89)	– (–)

Values in parentheses are standard deviation.

### Self-righting

All three species exhibited self-righting behaviour, but the amount and success of these actions varied between species. A full table of every successful self-righting trial can be found in supplementary data S2. *A. spinifera* was a prolific self-righter with most individuals engaging in the action of self-righting almost immediately upon inversion. With *A. spinifera*, when a self-righting event was engaged it was always successful. This was quite different from *T. s. scripta, w*here individuals were extremely reluctant about performing a self-righting action, only doing so after prolonged periods of inaction and with no human experimenter present in the room. In addition, not all self-righting events were successful or occasionally these were taking multiple attempts to successfully complete the manoeuvre. *C. serpentina* represented a mid-point between the other two species regarding self-righting. Four individuals demonstrated quick and prolific self-righting like *A. spinifera*, while two individuals were much more reluctant taking longer to self-right, like the behaviours exhibited by *T. s. scripta.* However, unlike *T. s. scripta*, all self-righting events attempted by *C. serpentina* were successful. Uniquely amongst the hatchling species in this study *C. serpentina* used its elongated tail during self-righting in addition to the use of the neck.

**Figure 4:**
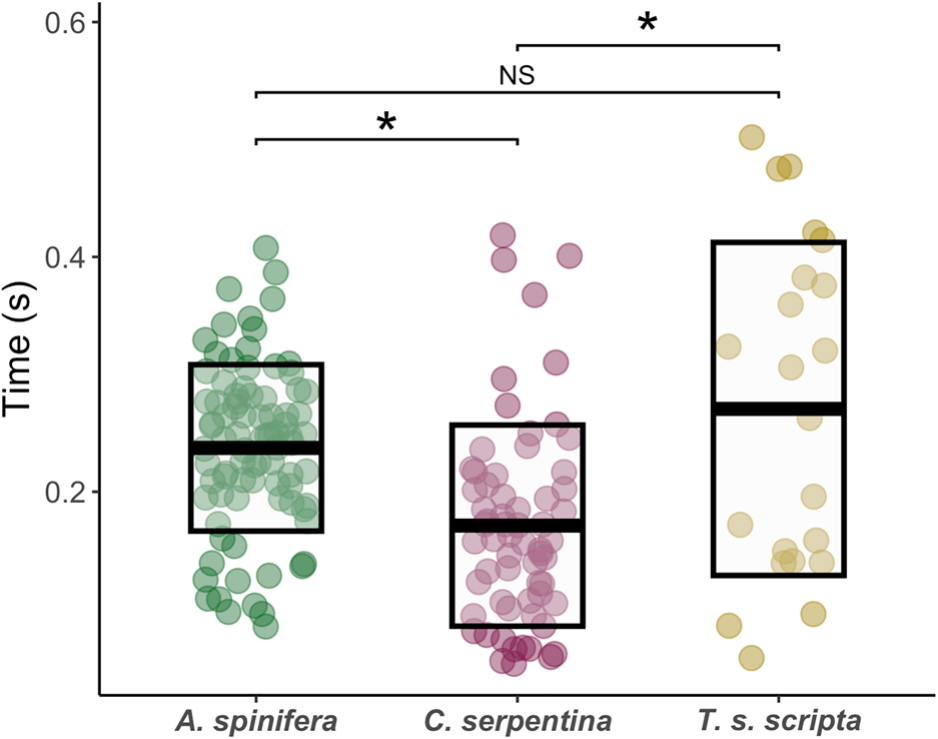
The time taken for the completion of a self-righting event in each species of hatchling. *C. serpentina* was significantly faster than the other species (F_2, 10.65_ = 9.32, p = 0.0046). Each point represents a self-righting trial, solid bar represents mean value and boxes represent the standard deviation, * = p < 0.05.

All self-righting events were quick, well below one second in duration (Fig. 4). Linear modelling detected a significant effect of species on the time to self-right (F_2, 10.65_ = 9.32, *p* = 0.0046). Post-hoc comparisons revealed that the self-righting impulse of *C. serpentina*, lasting on average of 0.17 (SD 0.09) seconds, was significantly faster than *T. s. scripta* (t_15.70_ = − 3.892, *p* = 0.0036) at 0.27 (SD 0.14) seconds, and *A. spinifera* (t_7.23_ = 3.333, *p* = 0.0285) at 0.24 (SD 0.07). There was no significant difference in the time to self-right between *T. s. scripta* and *A. spinifera* (Fig. 4).

**Figure 5:**
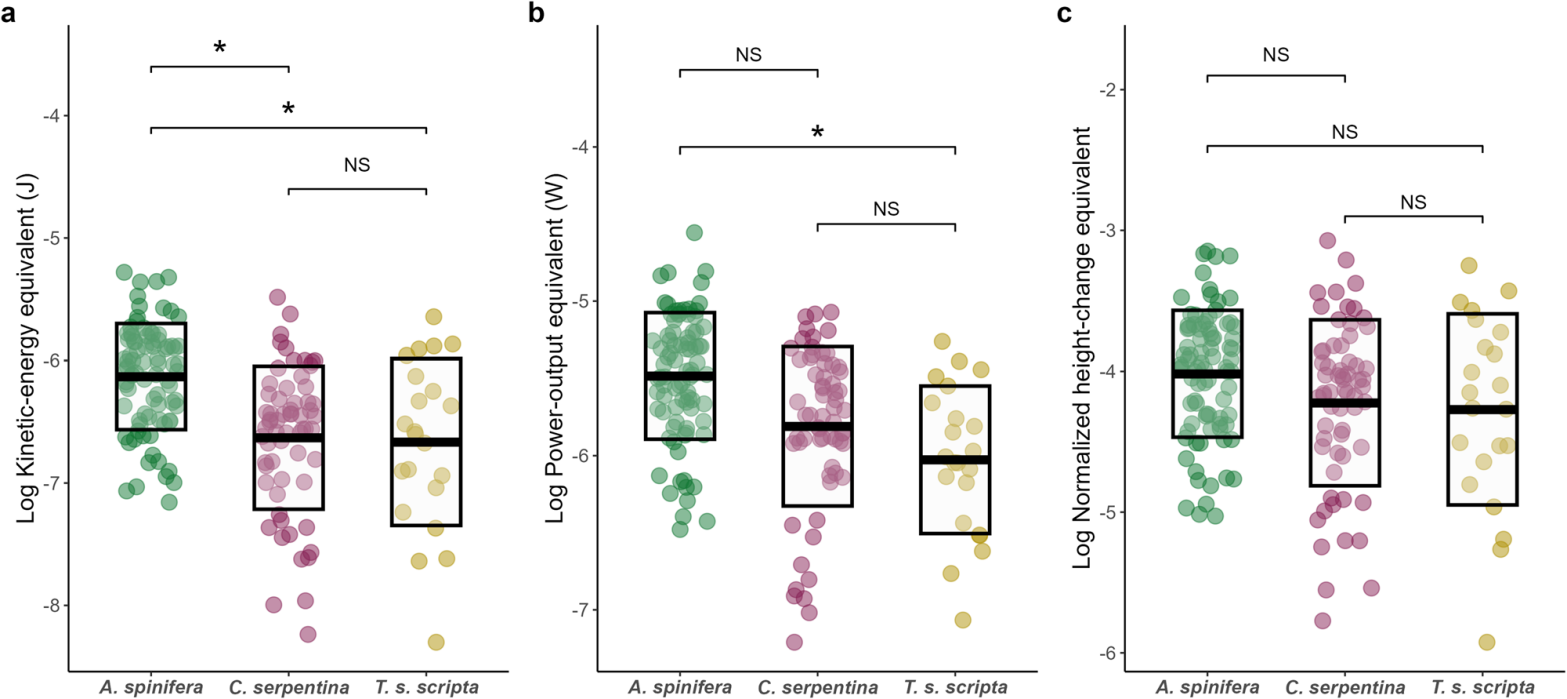
The biomechanical costs of self-righting in the three hatchling species (**a**) kinetic-energy equivalent, (**b**) power-output equivalent or (**c**) normalized height-change equivalent. Height-change equivalent was normalized to carapace width. Each point represents a self-righting trial, solid bars represent means, boxes denote the standard deviation and asterisk denotes significant differences between groups (* =  < 0.05).

The KEE of self-righting also differed significantly across the three hatchling species (*F*_*2, 11.60*_ = *7.61, p* = *0.0077*). Post hoc analyses demonstrated that *A. spinifera* exerted significantly more energy self-righting than *C. serpentina* (t_8.78_ = 3.57, *p* = 0.016) and *T. s. scripta* (t_13.90_ = 2.83, *p* = 0.034). There was no significant difference in the KEE of *C. serpentina* and *T. s. scripta* (Fig. 5a).

By looking at the PE of hatchlings (Fig. 5b) it is possible to understand the relationship between self-righting time and energetics. Power outputs were significantly different across species (*F*_*2, 11.55*_ = 6.09*, p* = 0.016). Post hoc analysis revealed that the PE of *A. spinifera* was significantly higher than *T. s. scripta* (t_13.38_ = 3.17, *p* = 0.019). However, there was no significant differences in PE between *C. serpentina* and either *A. spinifera* or *T. s. scripta* (Fig. 5b).

Figure 5c displays the normalized HE for each species of hatchling. The energy efficiency of self-righting did not differ between the hatchling species (*F*_*2, 12.12*_ = 1.35*, p* = 0.30). Despite requiring higher levels of KEE and PE for self-righting *A. spinifera* has similarly efficient self-righting when estimates of energetics were normalised to mass and shell shape.

## Discussion

### Self-righting time

The time taken to perform a self-righting action (Fig. 4) was generally fast in all three species, being well below a second in duration. Due to the high levels of predation in hatchlings, self-righting times are predicted to be fastest in this age class^18^. *C. serpentina* was the fastest self-righter in our study (Fig. 4). In previous work on *C. serpentina* where self-righting time was compared across turtles of varying ages, the time to self-right was significantly correlated with body mass (used as a proxy for age). Younger members of *C. serpentina* self-righted quicker than older members. The average self-righting time for all turtles under 1kg (range 0.927–0.254) was 0.69 (SD 0.23) seconds^7^. Although higher than the 0.17 (SD 0.09) seconds recorded in this study, it must be remembered that the smallest turtle in Ruhr et al.^7^ was 0.254 kg, in comparison the average weight for *C. serpentina* hatchlings in our study was 0.012 kg (Table 1). Therefore, the corresponding reduction in time to self-right is expected given the differences in turtle mass between studies. There is scant information about the self-righting times in hatchlings of both *A. spinifera* and *T. s. scripta* but the alignment of timings for *C. serpentina* between the current study and that of Ruhr et al.^7^ imbues confidence in our results.

*C. serpentina,* was the only species of turtle to reliably engage their tail in the act of self-righting. By acting as a second propulsive limb and directing the longitudinal rotation from both the anterior and posterior simultaneously, the long tails of *C. serpentina* hatchlings, have previously been demonstrated to speed up the righting process^19^*.* Given the similarities in shell shape and the reported similarities in neck proportions between *Chelydra* and *Trachemys*^7,32^, it seems reasonable to assume that the use of the tail could be responsible for the significantly faster righting speeds of *C. serpentina* observed in our study.

The significantly higher flatness and lower sphericity exhibited by *A. spinifera* should be indicative of slower righting times than both the other turtle species, when in fact there was no significant difference between the self-righting times of *A. spinifera* and *T. s. scripta.* Faster self-righting speeds in *A. spinifera* could be the result of their longer necks which provide the vertical force for self-righting^33,34^. Alternatively, the similarity in self-righting times between *A. spinifera* and *T. s. scripta* could be the result of *T. s. scripta* taking longer than predicted from shell shape to self-right. This may be indicative of a more “passive” response to predation relying on the mechanical protection of their more complete shells to avoid being eaten^14,35^. Studies have shown that red eyed sliders (*Trachemys scripta elegans*), an invasive species in Europe spend more time hiding within their shells in response to predation, when compared to the native Spanish terrapin (*Mauremys leprosa*). This reliance on a more passive hiding predator response has been attributed to the greatest danger facing *Trachemys* being from aquatic predators in their natural habitat. It is therefore safer to remain hidden in their shells when inverted terrestrially rather than quickly self-right and escape to the water^35^. An interesting avenue of future research would be mechanically characterising shell material and shape in these three species and quantifying their defensive capabilities. Given our results it seems reasonable to conclude that shell shape alone is not a reliable predictor of self-righting time in freshwater hatchlings and other anatomical features, such as the tail or neck and even the wider ecological contexts of the hatchlings must be considered.

### Self-righting effort

As with self-righting time the biomechanical effort required to self-right is thought to be correlated with shell shape in adult turtles^1,8^. Our hatchling results lend only partial support for these predictions. As predicted from their hydrodynamically shaped shells *A. spinifera* required more kinetic energy (Fig. 5a) to complete self-righting than the other two species. However, *A. spinifera* only exhibited a significant higher PE than *T. s. scripta* (Fig. 5b). While the modest increases in KEE and PE produced by *A. spinifera* could result from flatter and less spherical shell shapes, they could equally be due to the larger size and increased mass off the *A. spinifera* hatchlings (Table 1). Luckily, the HE metric normalizes KEE to the mass and shell width allowing comparison across species despite differences in mass and shell width.

In all species the mean value of HE (*A. spinifera* = 0.00015, *C. serpentina* = 0.00012, *T. s. scripta* = 0.00012) was considerably lower than previously reported HE values for self-righting in *C. serpentina*^7^ (HE mean = 0.30), indicating that regardless of species the act of self-righting is highly energy efficient in hatchlings, although nonidentical methodologies could also contribute to the lower HE seen in this study. This result confirms what was predicted by previous research into self-righting mechanics; where smaller turtles self-righted with increased efficiency compared with their larger counterparts^7^. Interestingly, and contra to our predictions based on shell shape alone, there was no significant difference in HE across all three species (Fig. 5c); meaning that accounting for mass and shell width the estimated energy needed to self-right was the same in all species. How then does *A. spinifera* maintain a relatively efficient self-righting action given the challenges of self-righting with a flatter carapace^8^? Several morphological features could account for the apparent improvements in energy efficiency. The extremely long neck of *A. spinifera*, sometimes as long as the carapace length^33^, will allow these turtles to increase the perpendicular distance between the applied force and the centre of rotation. This increase in the moment arm will mean that for a given force *A. spinifera* can produce a proportionally higher moment of rotation than the other two species with proportionally shorter necks. This potentially improves the precision of the righting action so that unnecessary movement of the centre of mass is minimised. Longer necks have already been associated with increased self-righting success in *Emydura subglobosa*^2^ and disproportionally shorter necks in older members of *C. serpentina* has been hypothesized as the reason for reduced self-righting energetic efficiency in larger animals. Our results also suggest that long necks may facilitate energetically efficient self-righting in *A. spinifera*.

In addition to long necks members of the Family Trionychidae, the soft-shell turtles, are characterised by the loss of the external keratinous shields, a flexible bridge region of the shell and the loss of the bony peripherals and suprapygals; leaving a rubbery and flexible skin flap at the shell edge^23,24^. This means that although *A. spinifera* has a low and flat shell, unlike the other two species, it has higher degree of flexibility especially at its edges. It is possible that flexing the edges of the shell during the self-righting action may help direct shell positioning or even facilitate raising the centre of mass during the turtle rotation. The plywood-like arrangement of tissues in the shells of trionychids would provide a flexible but strong material suited to such a function. It has already been suggested that the movable flexible flaps at the edge of the shell facilitate fast swimming bursts^24^. However, more work is needed on shell kinematics and material properties before more concrete conclusions can be drawn about the use of flexible shells as an aid to self-righting but the hypothesis that shell flexibility may also aid in the fast burst-like action of hatchling self-righting is intriguing.

Height-change equivalence is only an estimate of self-righting energy efficiency. Actual values of height-change equivalence may be higher than estimated if the turtle does not choose the most efficient self-righting trajectory and/or the kinetic energy output remains substantial at or beyond maximal height^7^, a possibility that could be facilitated by a long appendage such as a neck or tail. However, even when acknowledging the limitations of the HE estimates, it is interesting to note that despite differences in shell morphology the relative effort required to self-right remains constant and low^7^ across morphologically diverse hatchling species. This result is to be expected given the higher mortality rates of the hatchling life stage where the ability to self-right quickly and efficiently is essential.

### Perspectives

Our results indicate that the predicted evolutionary trade-off between the hydrodynamics of a flat shell and the improved self-righting ability of more rounded carapaces is either not as stark as theoretical predictions indicate or side stepped by other morphological adaptations in freshwater hatchlings. Counter to our hypotheses, differing levels of protection afforded by shell completeness or the predicted biomechanical advantages offered by shell curvature had little to no effect on the estimated biomechanical costs of self-righting. Although shell shape undoubtably contributes to the righting action, the differences in righting time and relative uniformity in biomechanical effort seen between species with similar and divergent shell shape indices, implies that other morphological factors may be influencing this ability.

Shell shape is likely only one of a suite of morphological features that may facilitate self-righting in turtles. At least in hydrodynamic turtles, any theoretical advantage imbued by shell shape does not translate to any significant time savings or estimated energetic efficiencies when hatchlings self-right. We theorise those adaptive features such as long necks, tails, or possibly flexibility in the carapace, ensure that costs of self-righting are minimal in freshwater turtle hatchlings. In light of our data we would advise against relying only on shell shape metrics as a proxy for self-righting ability, as has been done in the past^1,16,21^. Instead, future work should use more detailed kinematics to understand to what extent shell shape contributes to self-righting efficiency and whether other adaptive morphological traits contribute to the action. Such work could identify adaptive strategies used to facilitate self-righting across a range of testudines but if combined with ontogeny could identify if and how such adaptive strategies may change as turtles move from vulnerable early life stages to more secure later ones.

### Supplementary Information

Supplementary Information 1.

Supplementary Information 2.

## Data Availability

The datasets analysed during the current study are available in the Dryad repository, 10.5061/dryad.05qfttf92.
