# Supplementary material for: Shell shape does not accurately predict self-righting ability in hatchling freshwater turtles
Source: Sci Rep. 2024 Feb 28;14:4919. doi: 10.1038/s41598-024-54191-w (PMC10902340; doi:10.1038/s41598-024-54191-w)

**Title:** Shell shape does not accurately predict self-righting ability in hatchling freshwater turtles.

**Authors:** Adam van Casteren^1^, William I. Sellers^2^, Dane A. Crossley II^3^, Leah M. Costello^1^ & Jonathan Codd^1*^

**Affiliations:**

^1^School of Biological Sciences, University of Manchester, Manchester M13 9PL, UK

^2^School of Natural Sciences, University of Manchester, Manchester M13 9PL, UK.

^3^Department of Biological Sciences, University of North Texas, Denton, TX, USA

*Author for correspondence Jonathan.codd@manchester.ac.uk

**Supplementary material S1**

**Data analysis**

The experimental set up composed of 17 turtles. A total number of 170 observations were conducted.

The hypothesis for testing is whether the biomechanical outputs of self-righting: Kinetic energy equivalence (KEE), Power output equivalence (PE) and Height change equivalence (HE) are different between the three species of turtle.

To test this, Linear Mixed Effects Model (LMM) were fitted to our data. In all cases the dependant variable was the biomechanical output, the fixed independent variable was the species of turtle and the individual was set as the random effect.

To ensure the residuals of the model were normally distributed the continuous dependant variables were logged.

**Model KEE**

Dependant variable = **“kee_j”**, fixed effect variable = **“species”,** random effect = **“turtle”**

**model_kee <- lmer(log(kee_j,10) ~ species + (1|turtle), data=data)**

**Model results**

**summary(model_kee)**

**
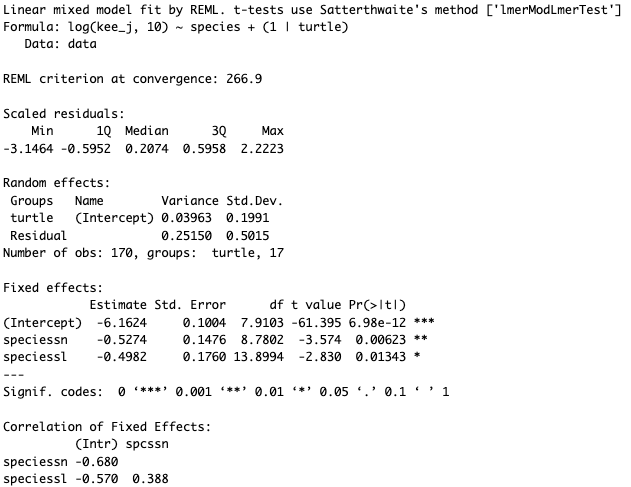
**

We used ANOVA (type II) followed by post hoc analyses (EMMs) to conduct pairwise comparisons between the three different species.

**anova(model_kee, type = 2)**

**
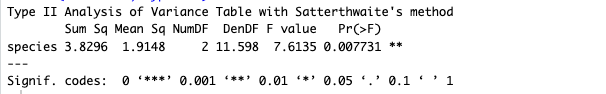
**

**emmeans(model_kee, list(pairwise ~ species), adjust = "tukey", lmer.df = "satterthwaite")**


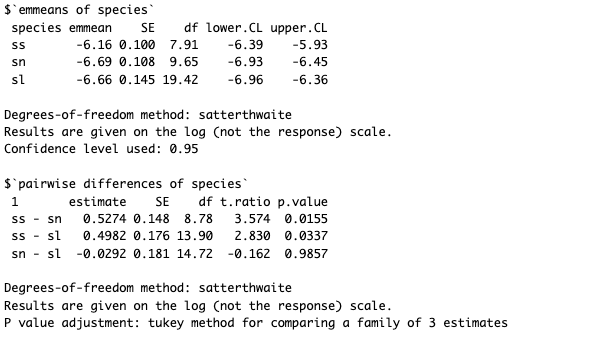


**Model PE**

Dependant variable = **“pe_w”**, fixed effect variable = **“species”,** random effect = **“turtle”**

**model_pe <- lmer(log(pe_W,10) ~ species + (1|turtle), data=data)**

**Model results**

**summary(model_pe)**


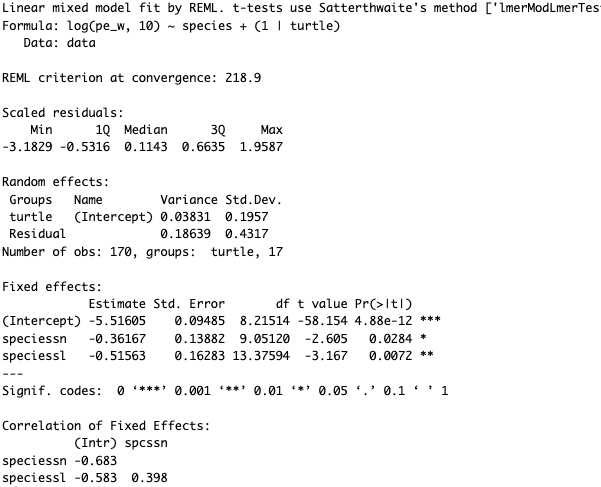


We used ANOVA (type II) followed by post hoc analyses (EMMs) to conduct pairwise comparisons between the three different species.

**anova(model_pe, type = 2)**


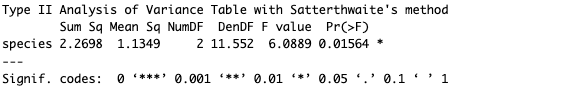


**emmeans(model_pe, list(pairwise ~ species), adjust = "tukey", lmer.df = "satterthwaite")**


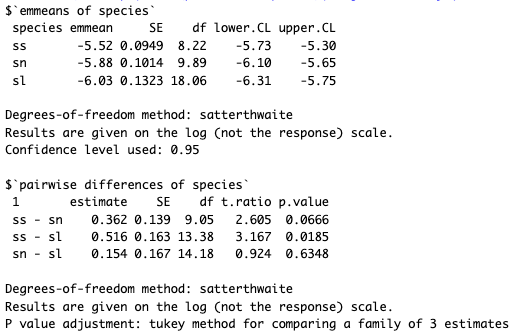


**Model HE**

Dependant variable = **“he”**, fixed effect variable = **“species”,** random effect = **“turtle”**

**model_he <- lmer(log(he,10) ~ species + (1|turtle), data=data)**

**Model results**

**summary(model_he)**

**
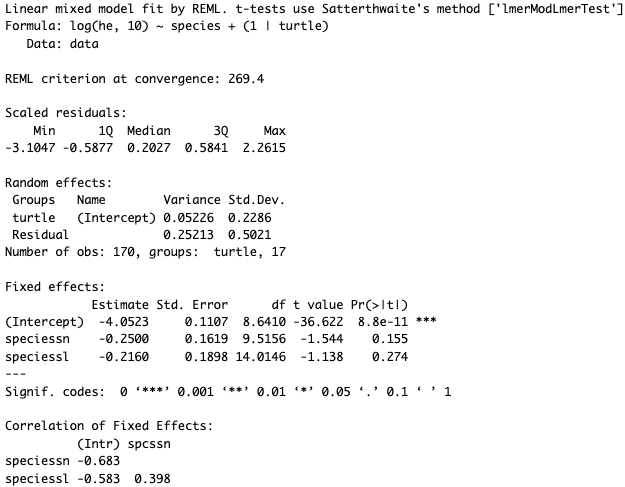
**

We used ANOVA (type II) followed by post hoc analyses (EMMs) to conduct pairwise comparisons between the three different species.

**anova(model_he, type = 2)**


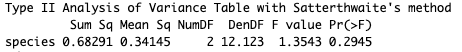


**Model Time**

Dependant variable = **“tot_time”**, fixed effect variable = **“species”,** random effect = **“turtle”**

**time <- lmer(tot_time ~ species + (1|turtle), data=data)**

**Model results**

**summary(time)**


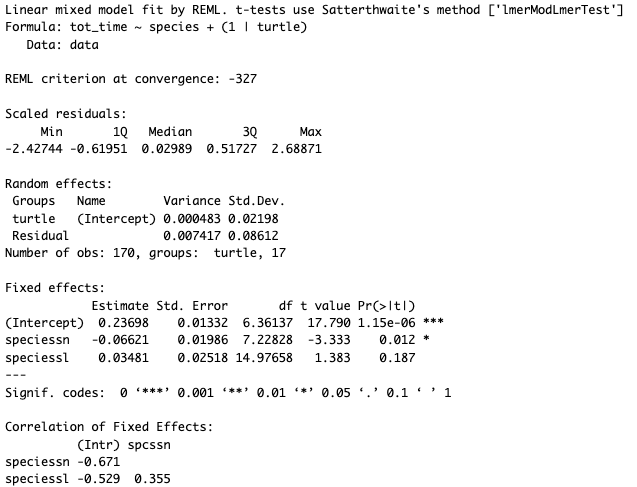


We used ANOVA (type II) followed by post hoc analyses (EMMs) to conduct pairwise comparisons between the three different species.

**anova(time, type = 2)**


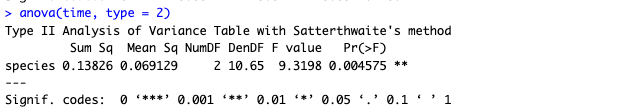


**emmeans(time, list(pairwise ~ species), adjust = "tukey", lmer.df = "satterthwaite")**


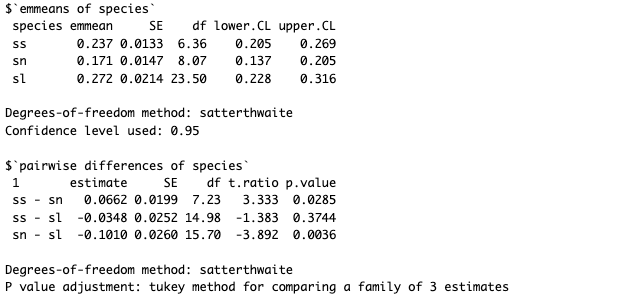

Supplement: Supplementary file 1 — Supplementary Information 1. [file 41598_2024_54191_MOESM1_ESM.docx]
